# Catastrophic thoracolumbar spinal massive hematoma triggered by intraspinal anesthesia puncture: A CARE-compliant case report

**DOI:** 10.1097/MD.0000000000017553

**Published:** 2019-10-11

**Authors:** Ruofeng Yin, Yuhang Zhu, Zhenbo Su, Pengyu Chang, Qingsan Zhu, Rui Gu, Hongjian Xing, Baolin Zhao, Yuan An, Fuwei Yang, Bo-Yin Zhang

**Affiliations:** a Orthopaedics Surgery Department; b Anesthesia Department, China-Japan Union Hospital of Jilin University; c Radiotherapy Department, First Bethune Hospital of Jilin University; d Hepatobiliary Department, China-Japan Union Hospital of Jilin University; e Neurosurgery Department, China-Japan Union Hospital of Jilin University, Changchun, Jilin Province, China.

**Keywords:** decompression, intradural hematoma, intraspinal anesthesia, spinal cord injury, spinal deformity

## Abstract

**Rationale:**

Intraspinal anesthesia, the most common anesthesia type of orthopedic operation, is regarded as safe and simple. Despite of the rare incidence, puncture related complication of intraspinal anesthesia is catastrophic for spinal cord. Here we present an intradural hematoma case triggered by improper anesthesia puncture. The principal reason of this tragedy was rooted in the neglect of spine deformities diagnosis before anesthesia. To the best of our knowledge, there is no specific case report focusing on the intradural hematoma triggered by improper anesthesia puncture.

**Patient concerns:**

Hereby a case of thoracolumbar spinal massive hematoma triggered by intraspinal anesthesia puncture was reported. The presenting complaint of the patient was little neurologic function improvement after surgery at 6-month follow-up.

**Diagnoses:**

Emergency MRI demonstrated that massive spindle-like intradural T2-weighted image hypointense signal masses from T12 to S2 badly compressed the dural sac ventrally, and his conus medullaris was at L3/4 intervertebral level with absence of L5 vertebral lamina. Hereby, the diagnoses were congenital spinal bifida, tethered cord syndrome, spine intradural hematoma, and paraplegia.

**Interventions:**

Urgent surgical interventions including laminectomy, spinal canal exploration hematoma removal, and pedicle fixation were performed. The patient received both medication (mannitol, mecobalamin, and steroids) and rehabilitation (neuromuscular electric stimulation, hyperbaric oxygen).

**Outcomes:**

Postoperation, he had regained only hip and knee flexion at II grade strength. His neurologic function was unchanged until 3 weeks postoperation. Six-month follow-up showed just little neurologic function improvement, and the American Spinal Injury Association grade was C.

**Lessons:**

By presenting an intradural hematoma case triggered by improper anesthesia puncture, we shared the treatment experience and discussed the potential mechanism of neurologic compromise. The principal reason for this tragedy is preanesthesia examination deficiency. Necessary radiology examinations must be performed to prevent misdiagnosis for spinal malformation.

## 1 Introduction

Intraspinal anesthesia, the most common anesthesia type of orthopedic operation, is regarded as safe and simple. Despite of the rare incidence, puncture-related complication of intraspinal anesthesia is catastrophic for spinal cord.^[1]^ It is generally known that spinal deformities are relative contraindication of lumbar puncture**.**^[2]^ However, the roughly normal back appearance of certain concealed spinal deformities, especially of the cord malformation often deceive anesthetist to perform intraspinal anesthesia. Under such circumstances, an improper puncture probably leads to iatrogenic neurologic damage such as intraspinal hematoma and spinal cord penetrating wounds. Nevertheless, careful physical examination and spine radiology inspection will be conductive to avert those mistakes. Noteworthy, once the neurologic detrimental intraspinal lesion was confirmed postpuncture, an urgent surgery intervention should be performed immediately.

Here we present an intradural hematoma case which triggered by improper anesthesia puncture. The principal reason of this tragedy was rooted in the neglect of spine deformities diagnosis before anesthesia. Furthermore, we share the treatment experience and discuss the potential mechanism of neurologic compromise.

## 2 Case report

**Figure 1:**
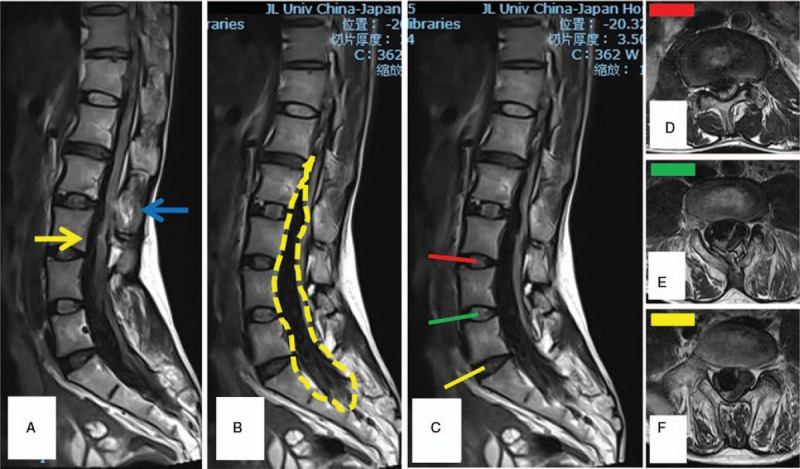
(A) The sagittal T2-weighted image (T2WI) magnetice resonance imaging (MRI) showed the conus medullaris was at L3/4 intervertebral level (yellow arrow). The anesthesia puncture site was at L2/3 intervertebral level with T2WI hyperintense signal on the interspinous ligament (blue arrow). (B) On T2WI MRI, massive spindle-like intraspinal T2WI hypointense signal masses spread from T12 to S2 ventrally (yellow dotted line). (C–F) The sagittal T2WI MRI showed the massive spindle-like intradural mass compress the spinal cord and cauda equina badly on each segments.

**Figure 2:**
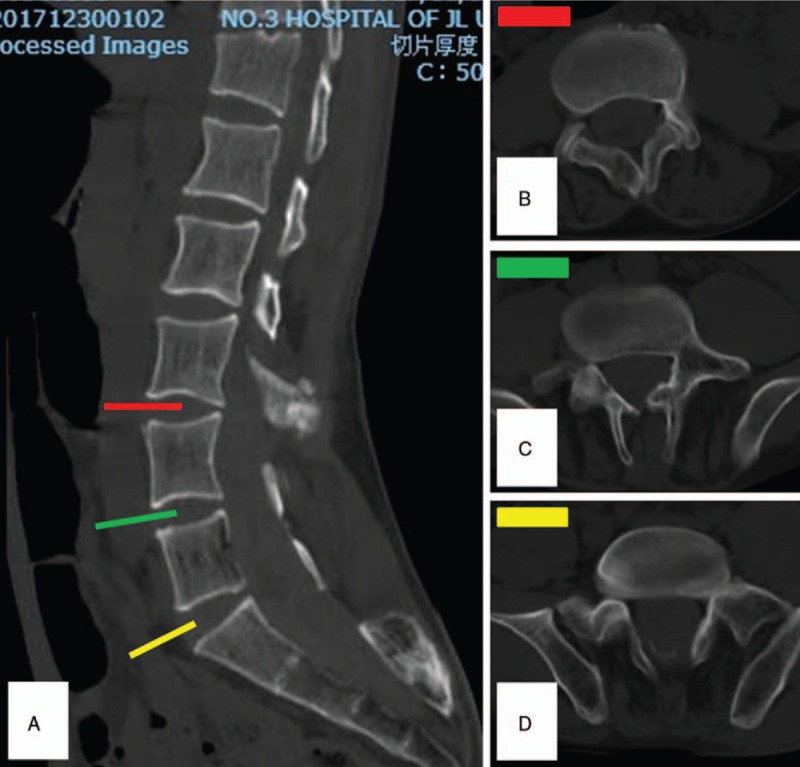
(A) The mid-sagittal reconstructed computed tomography (CT) showed the abnormal morphology of the vertebral bodies and spinous processes. (B–D) Axial CT demonstrated the vertebral laminae from L3 to L5 were more or less absent.

**Figure 3:**
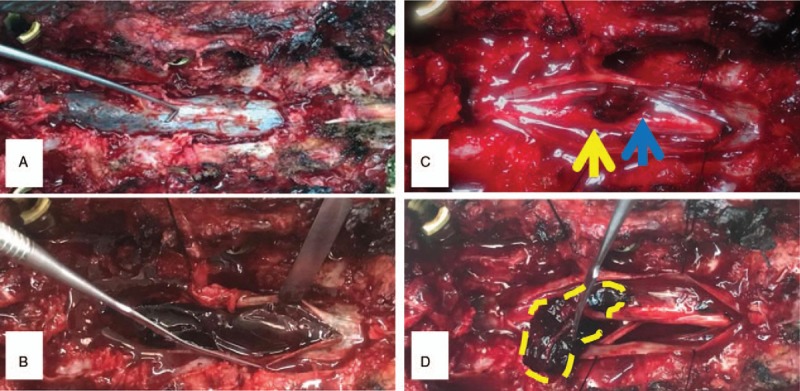
Post the T12 to S1 laminectomy, a tight dural sac was encountered. (A, B) By making a midline incision, several dark red rope-like blood clots spread across cauda equine were discovered. (C) Noteworthy, serveral needle-like penetrating points were found on the conus medullaris, and posterior artery on the surface was pierced. (D) The cauda equina was casted into clots which immobilized those nerves in the sac. Yellow arrow = conus medullaris, blue arrow = anesthesia puncture site, yellow dotted line = hematoma.

**Figure 4:**
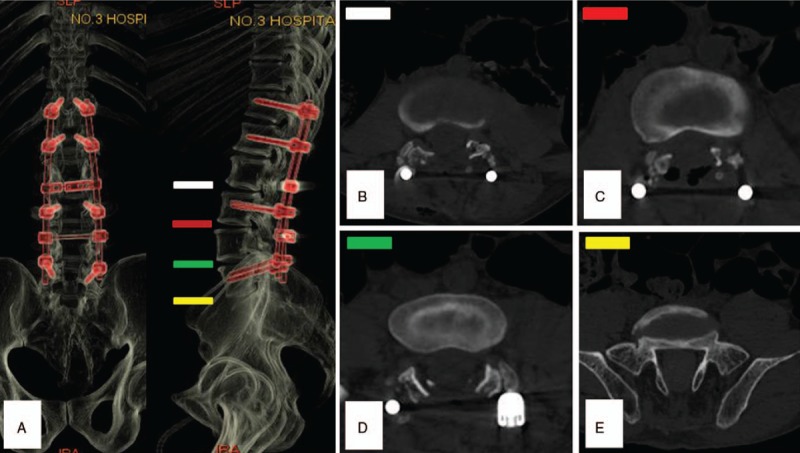
(A) The computed tomography (CT) volume reconstruction post OR. (B–E) Axial CT demonstrated decompression from L3 to L5.

A 35-year-old male underwent a metatarsophalangeal osteotomy with intraspinal anesthesia in grassroots hospital 4 days before admission. Unfortunately, his lower limbs motion and sensory were totally lost postoperation. According to his recollection, there were at least 4 low back punctures during anesthesia, and sometimes triggered leg numbness. The anesthetist claimed that he performed the back puncture repeatedly, but drains no cerebrospinal fluid (CSF). Physically, we found 2 pinhole scabs on the midline of L2 to L3 level. Still, there was a sparse tuft of hair on his lumbosacral skin. Additionally, he had bilateral 2 toes crossover deformity without hallux valgus. Neurologically, his lower extremities were paralyzed flaccidly from inguen level bilaterally, and the sphincter functions were completely deprived when admission. The ASIA grade was A. Both the blood count and coagulation markers were normal, and he denied any history of hematologic disease. Emergency magnetice resonance imaging (MRI) demonstrated that massive spindle-like intradural T2-weighted image (T2WI) hypointense signal masses from T12 to S2 badly compressed the dural sac ventrally (Fig. 1). Remarkably, his conus medullaris was at L3/4 intervertebral level, and L5 vertebral lamina was absent (Fig. 2). Thus, we made the primary diagnosis as congenital spinal bifida, tethered cord syndrome, spine intradural occupying lesion, and paraplegia. Consequently, an emergency OR was scheduled. After the T12 to S1 laminectomy, a tight dural sac was encountered. Then we made a posterior dural midline incision and found several dark red rope-like blood clots spread across cauda equine (Fig. 3). Of note, 3 needle-like penetrating points were found on the conus medullaris, and posterior artery on the surface was pierced (Fig. 3). Afterward, we gently evacuated intradural clots thoroughly, and then closed the sac with running suture (Fig. 4). The patient received both medication (mannitol, mecobalamin, and steroids) and rehabilitation (neuromuscular electric stimulation, hyperbaric oxygen). However, his neurologic function was unchanged until 3 weeks post-OR (Fig. 4). Afterward, he had regained only hip and knee flexion at II grade strength. Six-month follow-up showed just little neurologic function improvement, the American Spinal Injury Association grade was C.

## 3 Discussion

Intraspinal hematoma was a rare but severe complication following intraspinal anesthesia.^[3]^ Its reported incidence was 0.06% to 0.24% approximately.^[4]^ Etiologically, coagulopathy, spinal tumors, and musculoskeletal malformation were risky factors of anesthesia associated hematoma. Undoubtedly, an unreasonable anesthesia was the flashpoint of this disastrous case. Theoretically, a severe spine malformation per se is a relative contraindication of intraspinal anesthesia.^[4]^ The CT and MRI after admission showed multiple vertebral deformities (L5, S1) and a tethered spinal cord (Figs. 1 and 2). Unfortunately, visible evidences of him such as lumbosacral hair, secondary crossover toes deformity which was regarded as significant signs of neuromuscular system deformity were ignored during anesthesia evaluation. To avoid this situation, a detailed physical examination is indispensable before intraspinal anesthesia. Once spine malformations were suspected, examinations include MRI, CT, or elective spinal intraarterial angiography was required for diagnosis. Anatomically, the conus medullaris terminates at L1 inferior endplate in majority.^[5]^ However, the patient's tethered spinal cord makes the normal puncture site (L2–3) parallel to conus medullaris (Fig. 1). Moreover, a tight filum terminale immobilized the cord, which decreases the escape capacity when needle attacking. Consequently, repeated spinal punctures will definitely created fatal spinal cord penetrating injuries which include spinal cord parenchyma and blood vessels.

The blood supply of spinal cord was in charge of the anterior and posterior spinal arteries, which were located in the subarachnoid space (SAS). Due to the blood pressure of those terminal vessels was lower than CSF pressure in SAS, a tiny stab lesion would not lead to massive hemorrhage or hematoma.^[3]^ However, iatrogenic repeated spinal punctures not only aggravated the vascular injury, but drained CSF into subdural vascular space. Base on the connector mechanism, the CSF leakage triggered the diffuse SAS hematoma formation.

According to the occurrence site, hematoma can be classified into extradural/subdural/subarachnoid hematoma and hematomyelia.^[6]^ Regardless of its type, an early diagnosis and treatment is vital for neurologic recovery. Although the anesthetics application will confuse the timely neurologic symptoms recognition, the concealment often lasts no more than few hours. Once any neurologic abnormal was discovered post intraspinal anesthesia, an emergency MRI is indispensible for trouble shooting. Due to intracellular methemoglobin enriched, early hematoma (3–7 days) exhibited T2WI hypointense signal on MRI, and so does this patient.^[7]^

Provided an intradural compression was identified post intraspinal anesthesia, an urgent surgical intervention must be performed to rescue neurologic function. Postmassive intradural hemorrhage, the cauda equina was casted into clots which immobilized those nerves in the sac. Consequently, the clots should be nibbled away gently instead of en bloc resection to avoid nerve avulsion. Dvorak et al suggested that the optimal timing of spinal cord injury surgery intervention is <24-hour postinjury.^[8]^ Progressive nerve sabotage postspinal cord mechanical or ischemic injury, including axon retraction bulb formation, degenerative demyelination, and glial proliferation, which further deteriorate neurologic disorders.^[9]^ Obviously, this patient has missed the best treatment opportunity when hospitalized. Even so, a completely decompression operation is still desirable to minimize neurologic damnification.^[3]^

## 4 Conclusion

In summary, we report an iatrogenic intradural hematoma case which triggered by intraspinal anesthesia puncture herein. It is a disastrous anesthesia complication to both patient and doctor. The principal reason for this tragedy is preanesthesia examination deficiency. We must bear it in mind that spinal malformation, such as congenital spinal bifida and tethered cord syndrome, was a contraindication of intraspinal anesthesia. Once was suspected, necessary radiology examinations must be performed to prevent misdiagnosis as well as troublesome. Provided intradural compression was discovered postorthopedic operation associated intraspinal anesthesia, an urgent decompression should be performed immediately to facilitate neurologic rescue.

## Author contributions

**Conceptualization:** Ruofeng Yin, Boyin Zhang.

**Data curation:** Baolin Zhao.

**Formal analysis:** Rui Gu, Baolin Zhao, Yuan An.

**Funding acquisition:** Rui Gu, Boyin Zhang.

**Methodology:** Zhenbo Su, Boyin Zhang.

**Project administration:** Ruofeng Yin, Yuan An, Hongjian Xing.

**Resources:** Zhenbo Su, Pengyu Chang, Hongjian Xing, Fuwei Yang.

**Software:** Fuwei Yang.

**Supervision:** Pengyu Chang, Qingsan Zhu, Fuwei Yang.

**Validation:** Ruofeng Yin.

**Writing – original draft:** Zhenbo Su, Pengyu Chang, Qingsan Zhu, Boyin Zhang.

**Writing – review & editing:** Yuhang Zhu.

Boyin Zhang orcid: 0000-0001-7000-9674.
